# A Multi‐Target Approach to Colitis: Ergothioneine Promotes Recovery by Restoring the Intestinal Barrier, Reducing Inflammation, and Remodeling the Microbiota

**DOI:** 10.1002/fsn3.72294

**Published:** 2026-09-10

**Authors:** Lin Wang, Yao Song, Yucong Wang, Zhixin Xie, Xihan Xu, Jianchun Han, Rongxu Liu

**Affiliations:** ^1^ College of Food Science Northeast Agricultural University Harbin China; ^2^ Heilongjiang Institute of Green Food Science Harbin China

**Keywords:** anti‐inflammatory, ergothioneine, gut microbiota, inflammatory bowel disease, mucosal barrier

## Abstract

Ergothioneine (EGT) is a naturally occurring sulfur‐containing histidine derivative with antioxidant and anti‐inflammatory properties. However, its protective role and potential mechanisms in intestinal inflammation remain insufficiently understood. The impact of EGT on colitis was assessed in the present study using two experimental systems: an LPS‐induced inflammatory model in Caco‐2 cells and a DSS‐induced colitis model in mice. In vitro, Caco‐2 cells were treated with LPS and different concentrations of EGT, followed by assessment of inflammatory cytokines and tight junction‐related markers using ELISA, qRT‐PCR, and immunofluorescence staining. In vivo, DSS‐induced colitis mice received low‐, medium‐, or high‐dose EGT, and disease activity, colon histopathology, serum inflammatory mediators, immunoglobulins, and gut microbiota composition were analyzed. EGT treatment attenuated LPS‐induced inflammatory responses in Caco‐2 cells and partially restored the expression and distribution of tight junction proteins, including ZO‐1, Occludin, and Claudin‐1. In DSS‐induced colitis mice, EGT alleviated body weight loss, reduced disease activity index scores, improved colon shortening and histological injury, and decreased serum levels of TNF‐α, IL‐6, IL‐1β, IgM, and IgA. Moreover, EGT partially reversed DSS‐induced gut microbiota dysbiosis. Notably, high‐dose EGT was associated with the enrichment of *Lachnospiraceae NK4A136* group and *Enterorhabdus*, suggesting that these taxa may contribute to EGT‐mediated microbiota remodeling. Collectively, these findings indicate that EGT may alleviate experimental colitis through coordinated regulation of intestinal barrier integrity, inflammatory responses, and gut microbiota composition, supporting its potential as a functional food ingredient for intestinal inflammatory disorders.

## Graphical abstract

In LPS‐induced Caco‐2 cells, EGT inhibits inflammatory cytokine overproduction and restores tight‐junction proteins. In DSS‐induced colitis mice, EGT alleviates colitis symptoms, lowers inflammatory mediators and immunoglobulins, and remodels gut microbiota. Collectively, EGT ameliorates experimental colitis by preserving intestinal barrier integrity, suppressing inflammation, and modulating gut microbiota.

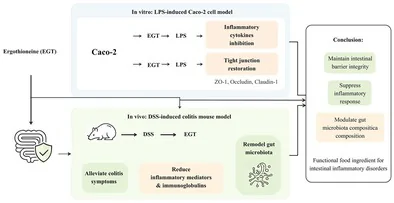

## 1 Introduction

Inflammatory bowel disease (IBD) is a condition characterized by inflammation of the intestines, with clinical symptoms primarily including abdominal pain, diarrhea, and weight loss (Gasparrini and Mazzoni 2024). It is closely associated with immune dysregulation and gut microbiota imbalance. IBD affects approximately 8 million individuals worldwide (Ng et al. 2017; Spiller et al. 2025). Beyond gastrointestinal distress, IBD is associated with high levels of psychological stress, depression, anxiety, and impaired sexual health, all of which further diminish wellbeing (Pandey et al. 2024; Romanukha et al. 2024). Moreover, the global prevalence of IBD is steadily rising, driven by accelerated urbanization and lifestyle changes. Mechanistically, many studies demonstrate that epithelial barrier dysfunction, imbalance of intestinal microbiota, and aberrant immune responses are central pathological features of IBD, preceding clinical onset and contributing to bacterial translocation, excessive immune activation, and chronic mucosal inflammation (Kwak et al. 2021; Ma et al. 2020; Wang, Lu, et al. 2024). Despite the proven efficacy of glucocorticoids and tofacitinib, their clinical application is limited by adverse effects, including immunosuppression, osteoporosis, hyperglycemia, and liver dysfunction (Qin et al. 2012; Louis and Flint 2017; Yu and Rodriguez 2017). Consequently, there is an urgent need for novel therapeutic strategies that effectively treat IBD while minimizing severe adverse reactions (Moon et al. 2024; You et al. 2021).

In recent years, natural dietary components have garnered increasing attention due to their significant potential in improving IBD. Ergothioneine (EGT), 2‐mercapto‐histidine trimethyl‐betaine, a natural histidine‐derived thiol compound and dietary antioxidant, was found in Claviceps purpurea by Chairles Tanret in 1909 (Asmus et al. 1996). In addition, a growing body of research has found that it is also widely present in mushrooms and vegetables (Halliwell et al. 2016; Marcinkowska and Jeleń 2022). Notably, EGT cannot be synthesized by the human body but can be obtained through dietary intake. After accumulating in human tissues, it helps reduce disease risks. Furthermore, extensive research indicates that EGT possesses anti‐inflammatory and antioxidant bioactivities and shows tremendous potential in regulating gut microbiota (Halliwell et al. 2018; Ey et al. 2007). EGT is found to be ubiquitously present in different organs, including the brain and small intestine. It can easily cross the blood—brain barrier after oral intake (Nakamichi et al. 2016; van der Hoek et al. 2022). Rats displayed no significant toxic effects in a 90 days toxicological investigation done by Marone et al. when tested at a high dose of 1600 mg/kg/day (Marone et al. 2016). Oral administration of EGT significantly diminishes inflammatory biomarkers and exhibits good oral bioavailability. Moreover it has been shown that EGT can improve CV disease (Williamson et al. 2020), liver damage (Dare et al. 2021) Alzheimer's disease (Roda et al. 2023) and avoid stone production (Mayayo‐Vallverdu et al. 2023; Liu et al. 2023), making it an alternative treatment for multiple chronic diseases. However, the potential role and mechanisms of EGT in improving IBD remain understudied.

This study systematically evaluated the ameliorative effects of EGT on IBD and its potential mechanisms using a lipopolysaccharide (LPS)‐induced Caco‐2 cell model and a dextran sulfate sodium (DSS)‐induced IBD mouse model.

## 2 Material and Methods

### 2.1 Material

Ergothioneine (2‐mercapto histidine trimethylbetaine, C_9_H_15_N_3_O_2_S, 99.7%) was purchased from MedChemExpress, Monmouth Junction, NJ, USA. The Caco‐2 human colon carcinoma cell line was obtained from Procell Life Science & Technology Co. Ltd. Wuhan, Hubei, China. LPS was purchased from Sigma, St. Louis, MO, USA. All other cell culture materials were purchased from VivaCell Bioscience, Shanghai, China, including fetal bovine serum (FBS), Dulbecco's modified Eagle's medium (DMEM), penicillin–streptomycin, trypsin, ethylenediaminetetraacetic acid (EDTA; without phenol red), Hanks' balanced salt solution (HBSS), and phosphate‐buffered saline (PBS).

### 2.2 Cell Culture

Caco‐2 cells were maintained in DMEM complete growth medium supplemented with 10% fetal bovine serum (FBS) and 1% penicillin–streptomycin solution (100 U/mL) at 37°C in a 5% CO_2_ atmosphere.

### 2.3 Identification of the Optimal Concentration of LPS

The effect of EGT on the survival rate of Caco‐2 cells was evaluated by the Cell Counting Kit‐8 (CCK8; Dojindo Laboratories, Japan) technique described by Noh et al. (Noh et al. 2022). The Caco‐2 cells were treated with LPS at varying concentrations (0.1, 1, 10, and 100 μg/mL, diluted in DMEM medium) for 4 h, respectively.

### 2.4 Cell Viability Assay

The effect of EGT on the survival rate of Caco‐2 cells was assessed using the CCK8 assay (Noh et al. 2022). Caco‐2 cells at the logarithmic growth phase were dispensed into 96‐well plates at a seeding density of 1 × 10^5^ cells/mL, with each well receiving 200 μL of the cell suspension. After 24 h of seeding, the cells were exposed to EGT at concentrations of 100, 200, 500, 1000, and 2000 μg/mL for 24 h, followed by treatment with LPS (100 μg/mL) for an additional 4 h. Subsequently, the supernatant was collected. Absorbance at 450 nm was measured using a microplate reader.

### 2.5 Quantitative Real‐Time Polymerase Chain Reaction (qRT‐PCR)

The experimental procedure was performed based on a previously described method (Xie et al. 2023). Briefly, Caco‐2 cells were cultured with EGT, either in the presence or absence of LPS (100 μg/mL). Total RNA was extracted from both colon tissue and Caco‐2 cells using TRIzol reagent, strictly following the manufacturer's instructions. Using a Transcription cDNA Synthesis Kit, the extracted RNA was subsequently reverse‐transcribed to generate complementary DNA (cDNA). Primer sequences are provided in Table S1.

### 2.6 Quantification of Inflammatory Factors (TNF‐α, IL‐1β, IL‐6)

The concentrations of cytokines in the cell culture supernatants were measured by enzyme‐linked immunosorbent assay (ELISA) kits (MULTI SCIENCES, Hangzhou, China) according to the manufacturer's instructions. Briefly, cell culture supernatants and serially diluted standard solutions were added to a 96‐well microplate precoated with specific capture antibodies against the target cytokines. Following incubation, unbound substances were removed by washing, and a biotinylated detection antibody was added to form an antibody–antigen–antibody sandwich complex. After another incubation and washing step, horseradish peroxidase‐conjugated streptavidin was added, followed by the addition of a chromogenic substrate. Absorbance was measured at a wavelength of 450 nm using a microplate reader.

### 2.7 Immunofluorescence Staining

After three PBS washes, cell fixation was carried out with 4% paraformaldehyde for 20 min at ambient temperature, followed by permeabilization using 0.5% Triton X‐100 for 15 min. The cells were then blocked for 1 h at room temperature with 5% BSA prepared in PBS. Subsequently, each well received 500 μL of primary antibody diluted 1:20 in 5% BSA, and the plates were kept at 4°C overnight. DAPI was used for nuclear counterstaining, after which the samples were mounted using an anti‐fade medium. Fluorescence imaging and analysis were finally performed on a fluorescence microscope.

### 2.8 Animal Models

Six‐week‐old male C57BL/6J mice were fed in a constant environmental system with a 12 h light/dark cycle, the temperature was 24°C ± 2°C, the relative humidity reached 50% ± 5% (Beijing Vital River Biotechnology Co. Ltd., Beijing, China). The water and standard feed were supplied without restriction (Beijing Harvest Feed Manufacturing Co. Ltd., Beijing, China). The ethical conduct of this study was approved by the Harbin Scientia Biotechnology Corporation Animal Ethics Committee (HRBSCIEC20250410). The research obeyed the guidelines in the National Institutes of Health Guide for the Care and Use of Laboratory Animals.

To assess the intervention efficacy of EGT in inflammatory bowel disease, colitis in mice was induced by 2.5% DSS (molecular weight 36,000–50,000 Da; MP Biomedicals, Solon, OH, USA). After 1 week of adaptive feeding, 60 mice were randomly divided into five groups (*n* = 12). The control group received daily gavage administration of 200 μL PBS (pH 7.2) during the 14 days intervention. The DSS group was given daily gavage doses of 200 μL PBS (pH 7.2) for 7 days. On day eight, the mice were administered water supplemented with 2.5% DSS for 7 days. Mice in EGT groups received EGT by gavage for 14 days, and 2.5% DSS was administered in drinking water from day 8 to day 14. Mice in the low‐dose EGT group (EGT‐L) received a daily gavage dose of EGT at a concentration of 30 mg/kg for 14 days. The medium‐dose EGT group (EGT‐M) was administered a daily gavage dose of EGT at a concentration of 75 mg/kg for 14 days. The high‐dose EGT group (EGT‐H) was administered a daily gavage dose of EGT at a concentration of 100 mg/kg for 14 days. On day 15, they were put to death. Blood samples were collected from the orbital venous plexus. The blood samples were centrifuged at 3000× *g* for 15 min at 4°C to obtain serum. Following euthanasia, the colon was excised, and after removal of the fecal contents, the colon tissues from each group were collected and rinsed with cold 0.85% physiological saline.

### 2.9 Assessment of Disease Activity and Histological Damage

The body weights of mice were recorded daily at 9:00 a.m. during the study. Stool blood content and fecal consistency were also assessed and documented on a daily basis. DAI was calculated based on body weight loss, stool consistency, and fecal blood (Wirtz et al. 2017). DAI scores range from 0 to 4.

### 2.10 Measurement of Cytokine and Immunoglobulin Levels

Commercial ELISA kits were used to measure the concentrations of inflammatory cytokines and immunoglobulins in serum.

### 2.11 H&E Staining of the Colon Tissue

Colon tissue samples were obtained from 12 randomly selected mice per group for histological examination. The collected tissues were fixed and preserved in 4% paraformaldehyde, embedded in paraffin, sectioned into 5‐μm thick slices, and subsequently stained with hematoxylin and eosin (H&E) (Jin et al. 2022). Histopathological evaluation was then performed using an Aperio Versa 8 microscope (Leica, Wetzlar, Germany).

### 2.12 16S rDNA Sequencing Analysis

After DNA extraction, the total DNA was propagated by polymerase chain reaction (PCR) (Wang et al. 2022) The PCR product was isolated and purified by AMPure XT Beads. Qualified PCR product was evaluated by Agilent 2100 Bioanalyzer and Illumina library quantification kits, then pooled and subjected to the Illumina NovaSeq 6000 platform. DADA2 was utilized to denoise the sequences and infer amplicon sequence variants (ASVs). Alpha and beta diversities were calculated by QIIME2, while relative abundance was applied to bacterial taxonomy classification. The Wilcoxon ranksum test was employed to detect genera exhibiting significant abundance variations using a significance threshold of *p* < 0.05. LDA effect size (LEfSe, LDA ≥ 3.0, *p* < 0.05) was performed using nsegata‐lefse.

### 2.13 Statistical Analysis

All quantitative data were first checked for completeness and consistency before statistical analysis. No data transformation was performed unless otherwise specified. Data are presented as mean ± standard deviation (SD). For comparisons involving more than two groups, a one‐way analysis of variance (ANOVA) was performed, followed by Tukey's honestly significant difference post hoc test for multiple comparisons. All statistical tests were two‐tailed, with the threshold for statistical significance set at *α* = 0.05. Statistical analyses were performed using GraphPad Prism 9.0, Statistical significance was defined as *p < 0.05 and **p < 0.01.

## 3 Results

### 3.1 Effect of LPS and EGT on Cell Viability of Caco‐2

**FIGURE 1:**
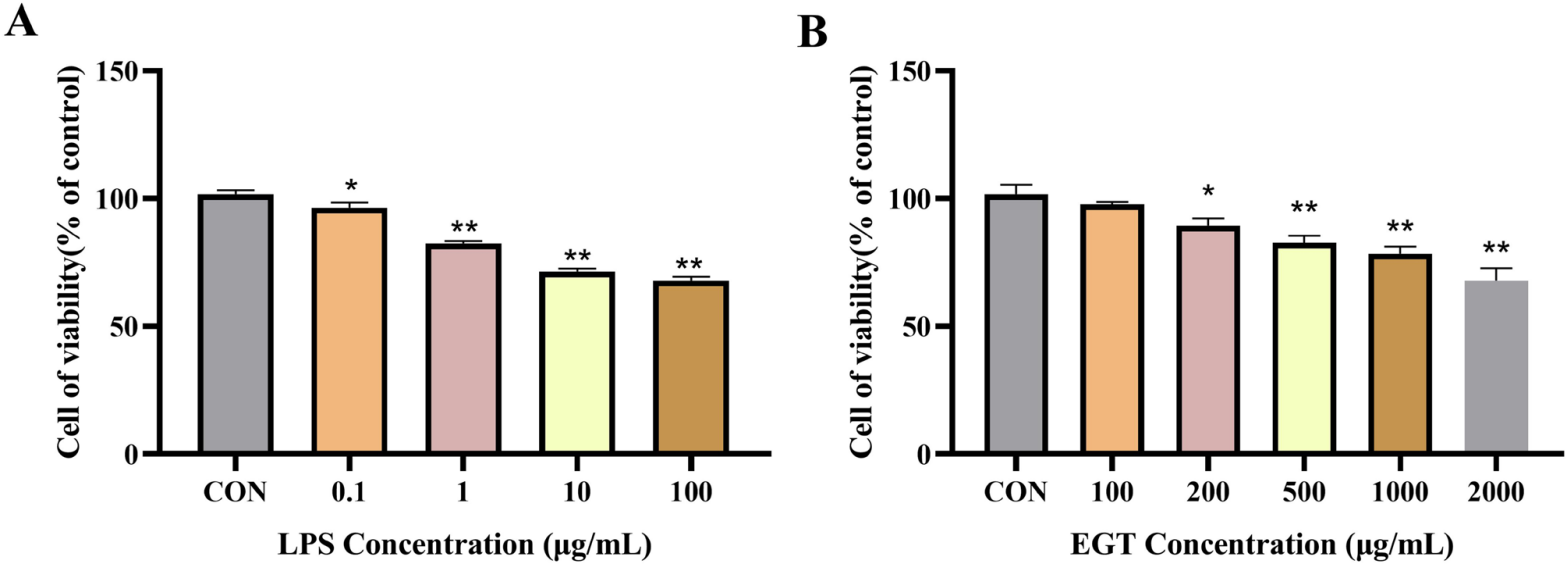
Effect of different concentrations of LPS on Caco‐2 cell viability (A) Effect of different concentrations of EGT on Caco‐2 cell viability (B) Data are presented as mean ± SEM (*n* = 3). *p < 0.05 and **p < 0.01 versus the CON group.

To determine the appropriate concentrations of LPS and EGT for subsequent experiments, the CCK‐8 assay was used to evaluate cell viability. As shown in Figure 1A, LPS treatment decreased Caco‐2 cell viability in a concentration‐dependent manner. Among the tested concentrations, 100 μg/mL LPS induced a marked but not complete reduction in cell viability, with cell viability remaining above the acceptable range for subsequent inflammatory stimulation experiments. Therefore, 100 μg/mL LPS was selected to establish an inflammatory injury model in Caco‐2 cells. Meanwhile, EGT at 100, 200, and 500 μg/mL maintained cell viability above 80%, whereas higher concentrations showed a greater inhibitory effect on cell viability. Based on these results, EGT concentrations of 100, 200, and 500 μg/mL were selected for subsequent experiments (Figure 1B).

### 3.2 Effects of EGT on Inflammatory Cytokine Production in LPS‐Treated Caco‐2 Cells

**FIGURE 2:**
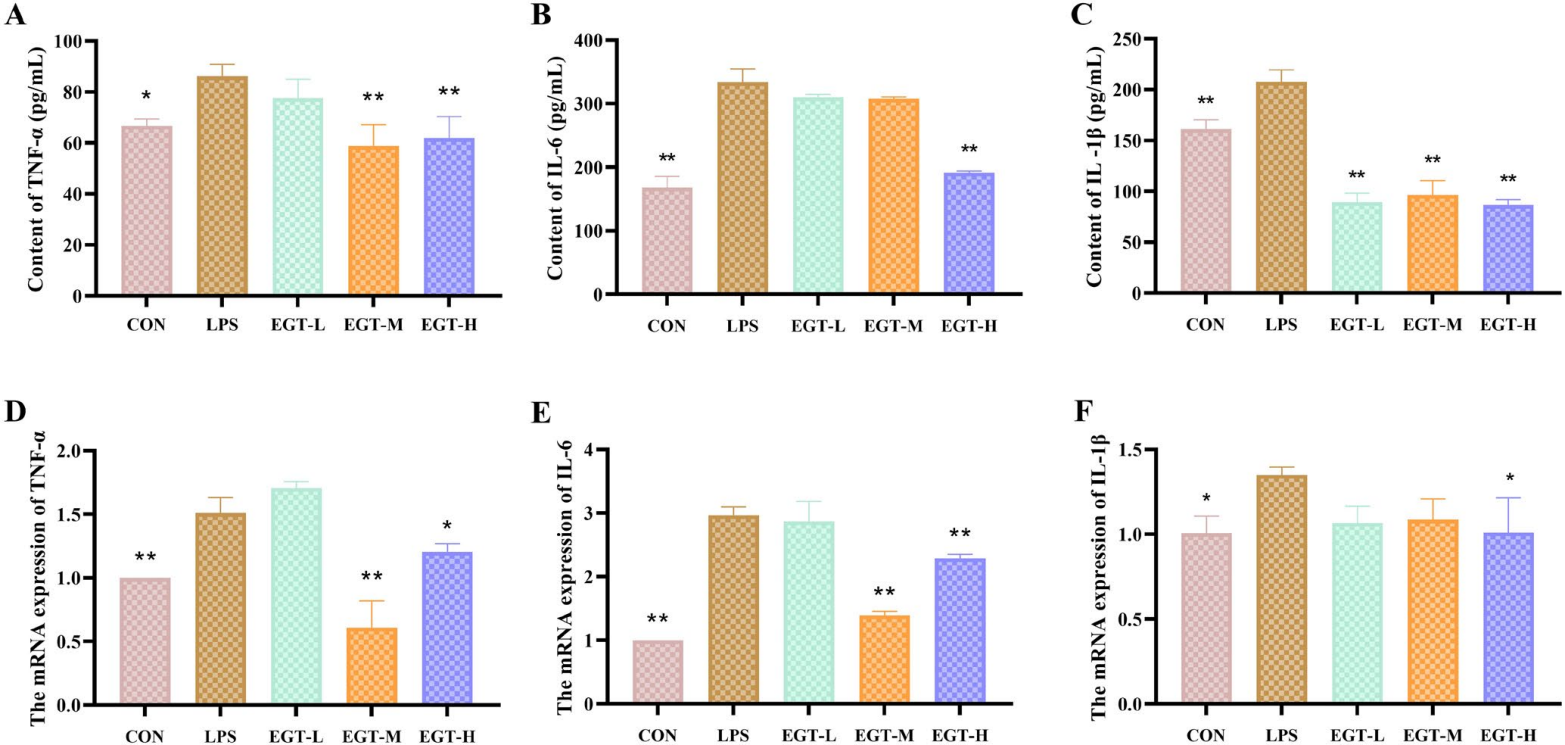
(A–C) ELISA was used to determine the levels of inflammatory cytokine. (D–F) qRT‐PCR showed the mRNA expression of inflammatory cytokine. CON: Cell with untreated; LPS: Cell treated with 100 μg/mL LPS; EGT‐L: Cell treated with 100 μg/mL EGT and 100 μg/mL LPS; EGT‐M: Cell treated with 200 μg/mL EGT and 100 μg/mL LPS; EGT‐H: Cell treated with 500 μg/mL EGT and 100 μg/mL LPS. Data are presented as mean ± SEM (*n* = 3). *p < 0.05 and **p < 0.01 versus the LPS group.

As shown in Figure 2A–C, LPS treatment significantly increased the levels of IL‐1β, IL‐6, and TNF‐α compared with the CON group. EGT treatment reversed this trend to varying degrees. Specifically, IL‐1β and TNF‐α levels were significantly reduced in the EGT‐M and EGT‐H groups, while IL‐6 levels were significantly decreased in the EGT‐H group compared with the LPS group.

Consistent with the ELISA results, qPCR analysis showed that LPS significantly upregulated the mRNA expression of TNF‐α, IL‐6, and IL‐1β (Figure 2D–F). EGT intervention attenuated these increases. Compared with the LPS group, the EGT‐M and EGT‐H groups exhibited significantly lower levels of IL‐6 and TNF‐α mRNA expression, with the EGT‐M group showing the most pronounced reduction. In addition, IL‐1β mRNA expression was significantly decreased in the EGT‐H group.

### 3.3 Effects of EGT on the Expression of Tight‐Junction Proteins In Vitro

**FIGURE 3:**
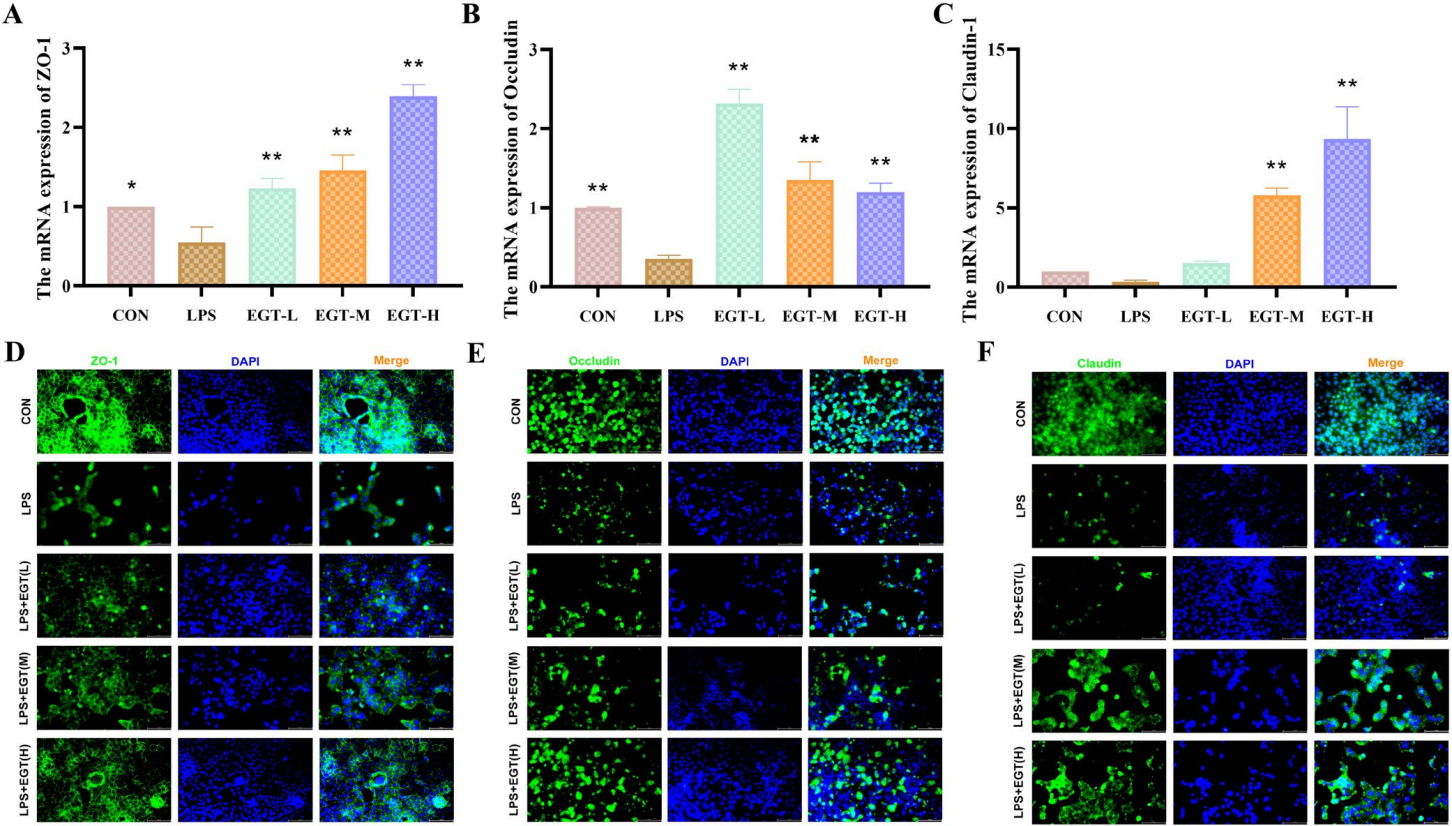
(A–C) The mRNA expression levels of ZO‐1, Occludin, and Claudin‐1 were quantified using qRT‐PCR. (D–F) Immunofluorescence staining of ZO‐1, Occludin, and Claudin‐1 in Caco‐2 cells. CON: Cell with untreated; LPS: Cell treated with 100 μg/mL LPS; EGT‐L: Cell treated with 100 μg/mL EGT and 100 μg/mL LPS; EGT‐M: Cell treated with 200 μg/mL EGT and 100 μg/mL LPS; EGT‐H: Cell treated with 500 μg/mL EGT and 100 μg/mL LPS. Data are presented as mean ± SEM (*n* = 3). *p < 0.05 and **p < 0.01 versus the LPS group.

Tight junction proteins (TJPs) play a vital role in preserving intestinal epithelial barrier integrity. To evaluate the effect of EGT on TJPs in LPS‐treated Caco‐2 cells, the spatial distribution of ZO‐1, Occludin, and Claudin‐1 was visualized by immunofluorescence microscopy. In the CON group, all three proteins displayed the most intense fluorescence signals, appearing as a continuous reticular pattern distributed between adjacent Caco‐2 cells and localized primarily at the cell–cell junctions (Figure 3D–F). In the CON group, the fluorescence signals of TJPs were either markedly weakened or absent, suggesting that LPS compromised TJP structure and disrupted epithelial barrier integrity. In contrast, EGT intervention significantly increased the fluorescence intensity of TJPs. Consistently, the mRNA levels of TJPs were determined. As shown in Figure 3A–C, compared with the CON group, LPS treatment significantly reduced the mRNA expression of ZO‐1, Occludin, and Claudin‐1. EGT treatment markedly reversed the LPS‐induced reduction in ZO‐1 and Occludin expression. Among the EGT‐treated groups, EGT‐L showed the highest Occludin expression, whereas EGT‐H exhibited the highest expression levels of ZO‐1 and Claudin‐1.

### 3.4 EGT Alleviates Clinical Symptoms in DSS‐Induced Colitis Mice

**FIGURE 4:**
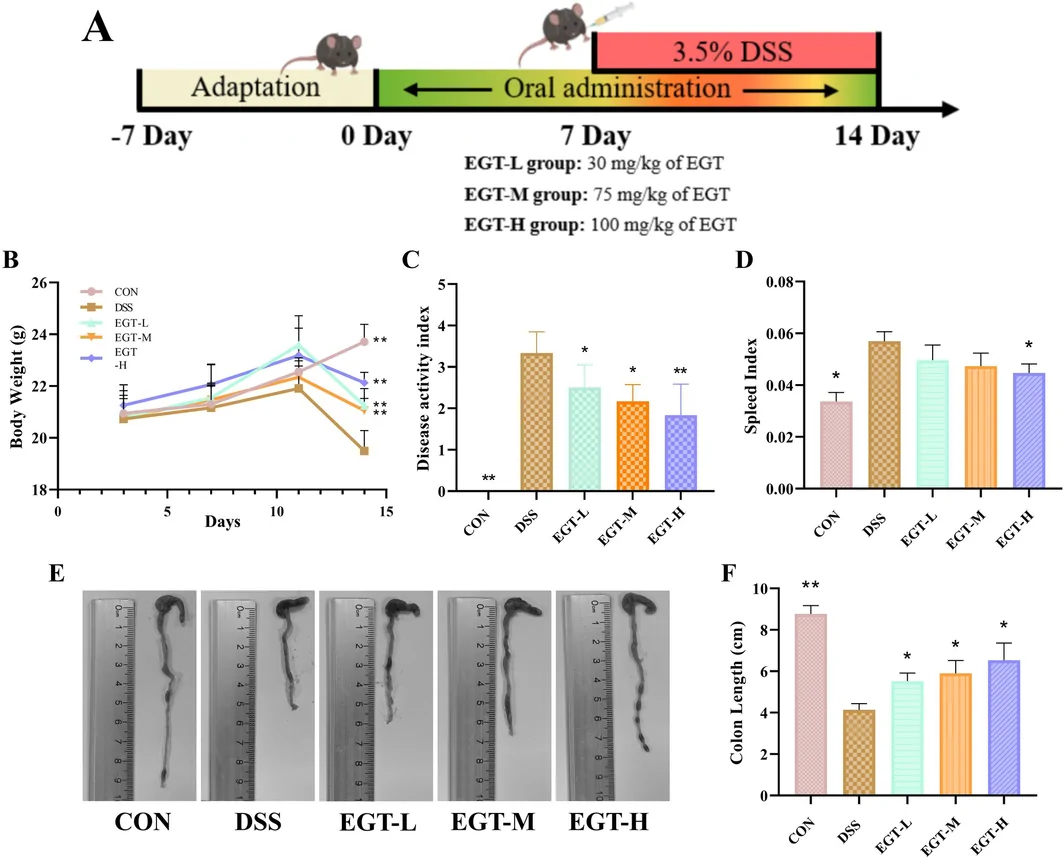
Impact of EGT on DSS‐Induced Colitis in Mice. (A) Experimental design: Mice were allocated into five groups: CON (untreated control), DSS (with 2.5% DSS induced), EGT‐L (DSS + 30 mg/kg/d EGT treatment), EGT‐M (DSS + 75 mg/kg/d EGT treatment), EGT‐H (DSS + 100 mg/kg/d EGT treatment). (B) Body weight and (C) DAI scores: Graphical depictions of alterations in body weight and DAI scores subsequent to DSS administration are presented. (D) Spheed Index. (E) Colon images (F) Measurements of colon length in mice from each group. Data are presented as mean ± SEM (*n* = 12). *p < 0.05 and **p < 0.01 versus the DSS group.

Daily body weight changes were recorded during the experimental period. Compared with the CON group, DSS‐treated mice showed obvious body weight loss beginning around day 11. EGT supplementation partially attenuated DSS‐induced body weight loss, with the EGT‐H group showing the most apparent improvement (Figure 4B). Consistently, DSS treatment increased the disease activity index (DAI), whereas EGT‐treated groups showed lower DAI scores compared with the DSS group (Figure 4C). DSS also increased the spleen index, while EGT supplementation reduced this increase to varying degrees, particularly in the EGT‐H group (Figure 4D). In addition, DSS treatment caused colon shortening, which was partially reversed by EGT administration, with the most notable effect observed in the EGT‐H group (Figure 4E,F). These results suggest that EGT supplementation alleviated, but did not completely prevent, DSS‐induced clinical symptoms of colitis.

### 3.5 EGT Attenuates Colon Tissue Damage in DSS‐Induced Colitis

**FIGURE 5:**
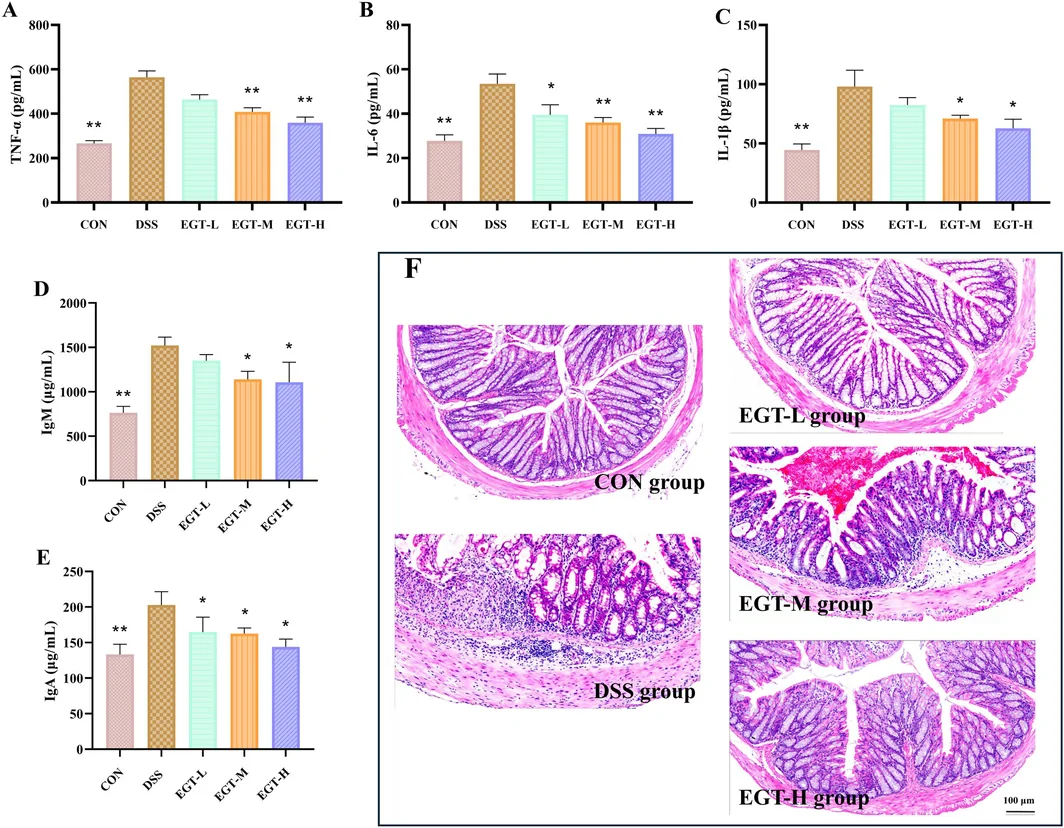
Effect of EGT on histopathological damage in DSS‐induced colitis. (A–E) ELISA was used to determine the levels of TNF‐α, IL‐6, IL‐1β, IgM and IgA. (F) Representative H&E‐stained images of colon sections from each experimental group are presented for histopathological analysis (magnification: 100×). CON: Untreated control; DSS: With 2.5% DSS induced; EGT‐L: 2.5% DSS + 30 mg/kg/d EGT treatment; EGT‐M: 2.5% DSS + 75 mg/kg/d EGT treatment; EGT‐H: 2.5% DSS + 100 mg/kg/d EGT treatment. Data are presented as mean ± SEM (*n* = 3). *p < 0.05 and **p < 0.01 versus the DSS group.

To further investigate the protective effects of EGT on colitis, this study used H&E staining to assess the morphological integrity and inflammatory status of colonic tissue. As shown in Figure 5F, the colonic tissue structure in the CON group was intact, characterized by an intact epithelial layer, regular crypt arrangement, and no obvious inflammatory cell infiltration. In contrast, mice in the DSS‐induced colitis model group exhibited severe pathological damage, including widespread destruction and shedding of intestinal villi, distorted crypt morphology, marked mucosal edema, and massive inflammatory cell infiltration in the lamina propria and submucosa. EGT intervention improved the aforementioned pathological changes in a dose‐dependent manner: in the EGT‐L group, mucosal structure was partially restored, inflammatory infiltration was reduced, and crypt morphology showed mild improvement; in the EGT‐M group, mucosal integrity was significantly restored, with more regular arrangement of villi and crypts and a marked reduction in inflammatory cell infiltration; notably, in the EGT‐H group, the colonic structure was nearly restored to normal, with minimal epithelial damage, well‐preserved crypt structure, and sparse inflammatory cell infiltration.

Pro‐inflammatory cytokines and immunoglobulins are key components in the pathogenesis of ulcerative colitis. In Figure 5A–E, compared with the CON group, DSS intervention significantly increased the serum levels of pro‐inflammatory cytokines including TNF‐α, IL‐6, and IL‐1β, as well as immunoglobulins IgM and IgA, indicating that DSS induced systemic inflammatory responses and excessive activation of intestinal mucosal immunity. After intervention with different doses of EGT, the levels of these pro‐inflammatory factors and immunoglobulins decreased in a dose‐dependent manner. Specifically, medium‐ and high‐dose EGT (EGT‐M, EGT‐H) significantly downregulated the expression levels of TNF‐α, IL‐6, IL‐1β, IgM, and IgA.

### 3.6 EGT Modulates the Gut Microbiota in Mice With DSS‐Induced Colitis

**FIGURE 6:**
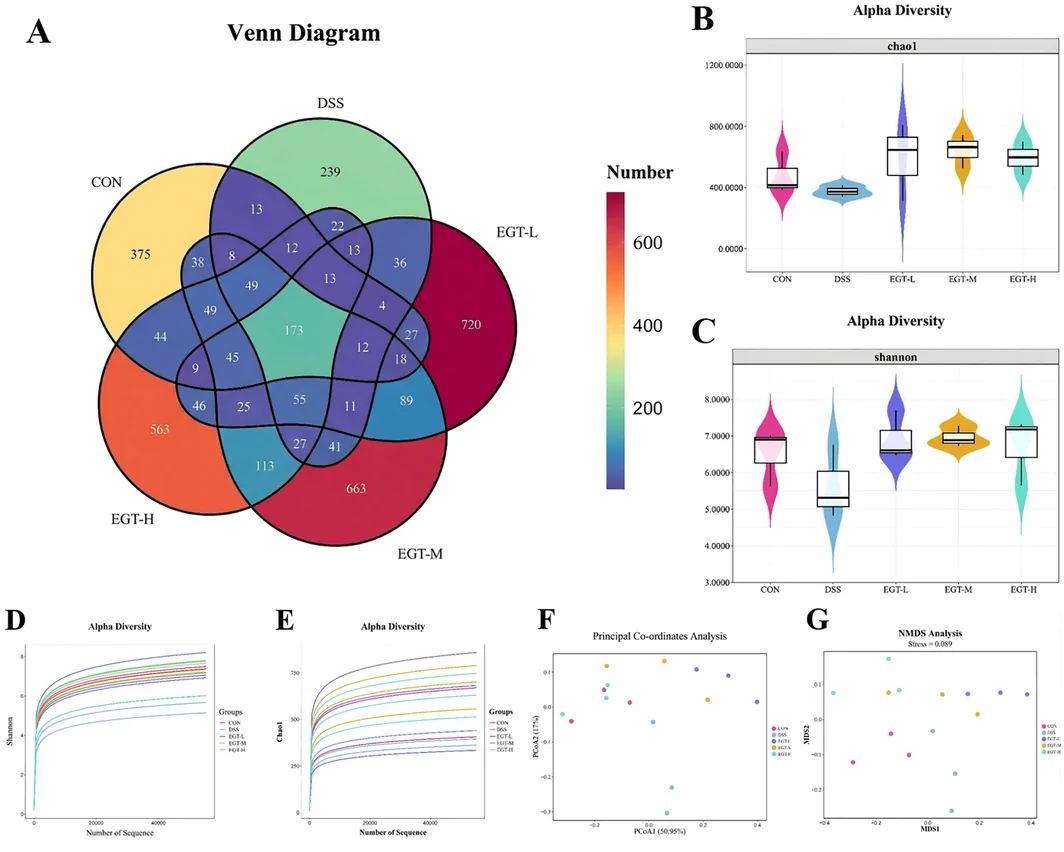
Effects of EGT on gut microbiota diversity and community structure in DSS‐induced colitis mice. (A) Venn diagram showing the shared and unique ASVs among different groups. (B) Chao1 index. (C) Shannon index. (D, E) Rarefaction curves. (F, G) Principal coordinates analysis (PCoA). CON: Untreated control; DSS: With 2.5% DSS induced; EGT‐L: 2.5% DSS + 30 mg/kg/d EGT treatment; EGT‐M: 2.5% DSS + 75 mg/kg/d EGT treatment; EGT‐H: 2.5% DSS + 100 mg/kg/d EGT treatment.

The effects of EGT on the gut microbiota in DSS‐induced colitis mice were investigated using 16S rRNA gene sequencing. A Venn diagram was used to visualize the numbers of shared and unique amplicon sequence variants (ASVs) among the different groups. The number of unique ASVs was lower in the DSS group than in the CON group. In contrast, the EGT‐L, EGT‐M, and EGT‐H groups showed increased numbers of unique ASVs compared with the DSS group (Figure 6A). Alpha diversity analysis showed that both the Chao1 and Shannon indices were reduced in the DSS group relative to the CON group, whereas EGT treatment increased these indices to varying degrees. Rarefaction curves approached saturation in all groups, indicating sufficient sequencing depth (Figure 6D,E). Beta diversity analysis based on PCoA revealed a clear separation between the DSS and CON groups, indicating that DSS markedly altered gut microbial structure. The EGT‐treated groups showed a trend toward the CON group, with the EGT‐H group appearing closest to the control (Figure 6F).

**FIGURE 7:**
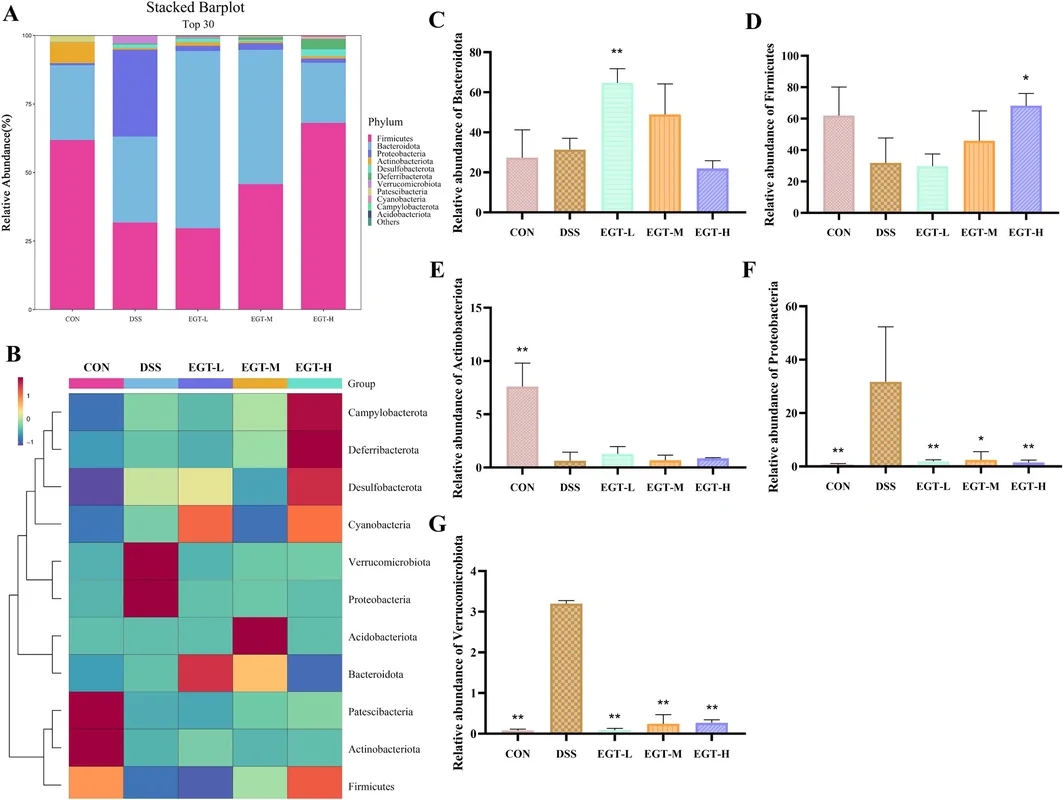
The effects of EGT on the composition of gut microbiota at the phylum level were evaluated in mice with DSS‐induced colitis. (A) Stacked bar plot showing the relative abundance of major phyla in each group. (B) Heatmap of the relative abundance of major phyla. (C) Bacteroidota. (D) Firmicutes. (E) Actinobacteriota. (F) Proteobacteria. (G) Verrucomicrobiota. CON: Untreated control; DSS: With 2.5% DSS induced; EGT‐L: 2.5% DSS + 30 mg/kg/d EGT treatment; EGT‐M: 2.5% DSS + 75 mg/kg/d EGT treatment; EGT‐H: 2.5% DSS + 100 mg/kg/d EGT treatment. Data are presented as mean ± SEM (*n* = 6). *p < 0.05 and *p* < 0.01 versus the DSS group.

*Firmicutes* and *Bacteroidota* were the dominant phyla in the CON group. Compared with the CON group, DSS treatment reduced the relative abundances of *Firmicutes* and *Actinobacteriota* while increasing those of *Proteobacteria* and *Verrucomicrobiota*. EGT treatment partially reversed these DSS‐induced changes. The EGT‐L group showed increased *Bacteroidota* and reduced *Verrucomicrobiota* and *Proteobacteria*. The EGT‐M group exhibited higher *Firmicutes* and *Bacteroidota*, together with lower *Proteobacteria* and *Verrucomicrobiota*. Similarly, the EGT‐H group showed increased *Firmicutes* and reduced *Proteobacteria* and *Verrucomicrobiota* while the abundance of *Bacteroidota* approached that of the CON group (Figure 7A–G).

**FIGURE 8:**
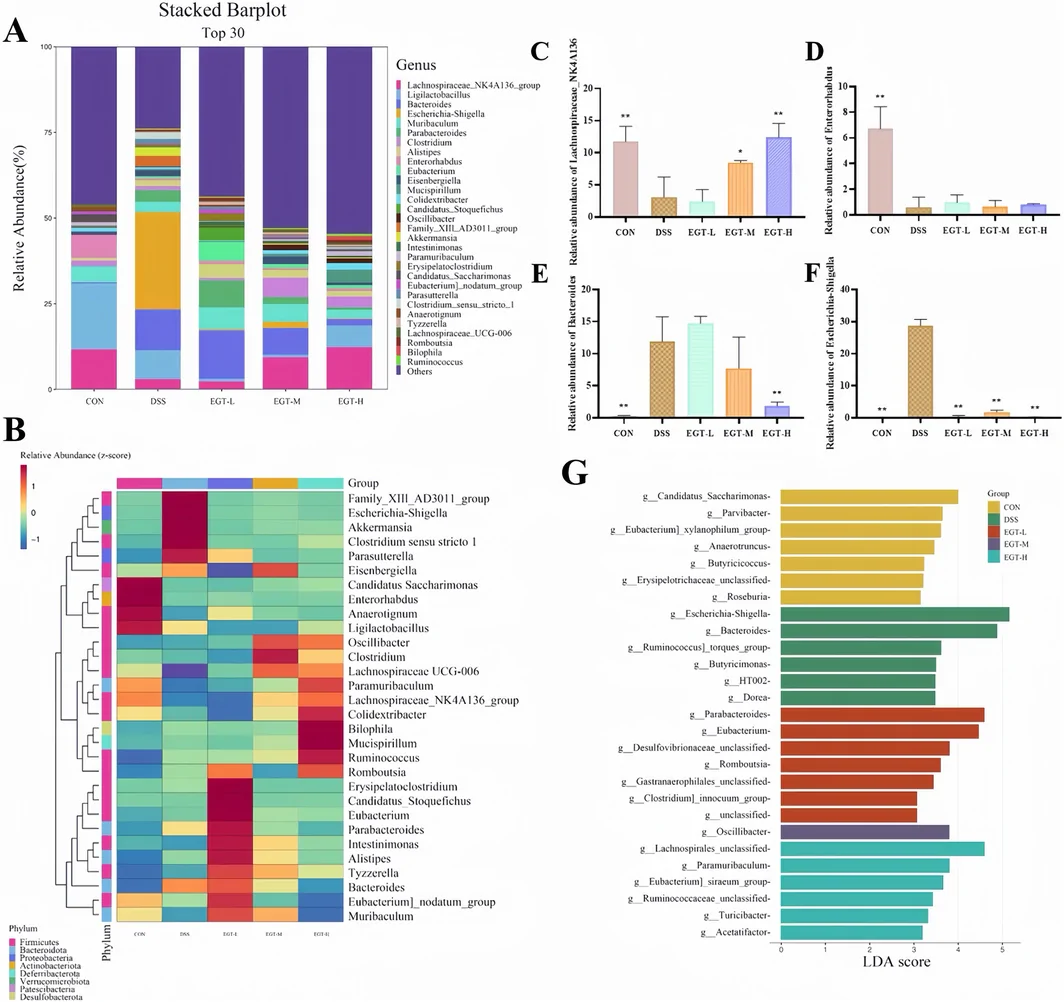
Impact of EGT on gut microbiota composition at the genus level in mice with DSS‐induced colitis. (A) Stacked bar plot showing the relative abundance of major genera in each group. (B) Heatmap of the relative abundance of major genera. (C) Lachnospiraceae NK4A136 group. (D) *Enterorhabdus*. (E) *Bacteroides*. (F) *Escherichia*‐*Shigella*. (G) LDA score histogram of differential taxa identified by LEfSe analysis. CON: Untreated control; DSS: With 2.5% DSS induced; EGT‐L: 2.5% DSS + 30 mg/kg/d EGT treatment; EGT‐M: 2.5% DSS + 75 mg/kg/d EGT treatment; EGT‐H: 2.5% DSS + 100 mg/kg/d EGT treatment. Data are presented as mean ± SEM (*n* = 6) for panels C–F. *p < 0.05 and **p < 0.01 versus the DSS group.

At the genus level, DSS treatment was associated with increased relative abundances of *Bacteroides* and *Escherichia‐Shigella* and a decreased abundance of *Lachnospiraceae NK4A136* group. EGT treatment altered these DSS‐induced changes to varying degrees. Compared with the DSS group, the EGT‐L group showed reduced *Escherichia‐Shigella* abundance, the EGT‐M group showed increased *Lachnospiraceae NK4A136* group abundance, and the EGT‐H group showed lower abundances of *Escherichia‐Shigella* and *Bacteroides*. Overall, these findings suggest that EGT partially reversed DSS‐induced alterations in genus‐level gut microbiota composition (Figure 8A–F).

LEfSe analysis further identified differential taxa among groups (Figure 8G). The CON group was enriched in *Candidatus Saccharimonas* and *Parvibacter*, whereas *Escherichia‐Shigella* was enriched in the DSS group. Different EGT doses were associated with enrichment of distinct taxa, including *Parabacteroides* and *Eubacterium* in EGT‐L, *Oscillibacter* in EGT‐M, and *Paramuribaculum* together with *Eubacterium_siraeum*_group in EGT‐H.

## 4 Discussion

This study aimed to elucidate the mechanism by which EGT modulates gut barrier function and alleviates intestinal inflammation. In recent years, the destruction of the intestinal epithelial barrier and the accompanying inflammatory response have been considered the fundamental pathological mechanism of UC (Scharl et al. 2009). Consequently, this study explores the regulatory effects of EGT on the intestinal inflammatory responses of LPS‐induced Caco‐2 cells in vitro, and demonstrates alleviation efficacy in a DSS‐induced murine colitis model.

The overexpression of inflammatory cytokines is closely associated with both the onset and progression of UC (Zhang et al. 2024). TNF‐α contributes to intestinal epithelial damage by disrupting the epithelial barrier (Wu et al. 2015). IL‐1β exacerbates intestinal inflammation through the promotion of granulocyte infiltration and the activation of innate immune cells (Kmieć et al. 2017). IL‐6 modulates pro‐inflammatory T‐cell subsets; its overexpression inhibits regulatory T cells, thereby intensifying inflammatory responses (Lee et al. 2024; He et al. 2024). Importantly, EGT treatment dramatically downregulated proinflammatory marker expressions (qPCR and ELISA). In fact, it has been shown that EGT can alleviate LPS stimulation in the Caco‐2 cell line. EGT inhibits inflammation through upregulating antioxidant transcription factor expression and decreasing TNF‐α and IL‐1β mRNA expression found by Salam, which are in agreement with the present work (Salama et al. 2021).

The intestinal mucosal barrier system has an important function to maintain the intestinal epithelial barrier integrity against pro‐inflammatory mediators, and consists of mainly three parts: the mucus layer, the epithelial cell layer, and the lamina propria. Compromised barrier function facilitates the translocation of pathogens and toxins across the epithelium, inducing inflammation, aggravating the course of the disease (Capaldo et al. 2017).

Tight junction proteins serve as critical molecular markers for maintaining structural and functional integrity of the epithelial barrier (Mayangsari and Suzuki 2018; Zhou et al. 2017; Liu et al. 2024). The findings indicate that LPS treatment significantly downregulated the mRNA expression levels of ZO‐1, Occludin, and Claudin‐1. It should be noted that the effects of EGT on tight junction‐related markers were not strictly dose‐dependent. For example, EGT‐L showed the strongest effect on Occludin mRNA expression, whereas EGT‐H induced higher ZO‐1 and Claudin‐1 expression. This discrepancy may reflect the different regulatory sensitivity of individual tight junction proteins to EGT treatment. Tight junction assembly is a dynamic process involving multiple structural and scaffold proteins, and different components may respond differently to antioxidant or anti‐inflammatory stimulation (Rawat et al. 2025; Jiang et al. 2026). Therefore, the present results suggest that EGT may regulate intestinal barrier function through multiple targets rather than through a simple linear dose–response pattern. Immunofluorescence results further demonstrated that EGT treatment improved the expression of tight junction proteins. In the previous studies, it was shown that EGT effectively alleviates oxidative damage in Caco‐2 cells and maintains cellular homeostasis through its potent antioxidant properties, thereby indirectly preserving the expression and function of tight junction proteins. These results are consistent with the present study (Tian et al. 2023).

In the present study, 100 μg/mL LPS was selected to establish the inflammatory injury model in Caco‐2 cells because it induced a clear inflammatory response while maintaining a measurable level of cell viability. However, this concentration also reduced cell viability, suggesting that part of the observed response may be related to cellular injury rather than inflammation alone. Therefore, the protective effects of EGT observed in vitro should be interpreted cautiously. Future studies should include lower LPS concentrations or additional inflammatory models, such as TNF‐α‐ or IL‐1β‐induced epithelial inflammation, to distinguish anti‐inflammatory effects from protection against cytotoxic injury more precisely.

According to these encouraging in vitro results, we used an induced DSS‐colitis model for studying intestinal regulation effect by EGT. Model group showed typical disease phenotype, including lower colon length, an elevated spleen index, massive bodyweight loss, and higher disease activity index (DAI). However, with EGT Supplement significantly reduced those changes were observed, reflected in the marked improvement on the loss in colon length, improved DAI score, a reduction in the spleen index, and decreased pathological grade in the mucosa tissue (Lin et al. 2024). The antiinflammatory and protective barrier effect of EGT in vivo was confirmed directly by histological analysis. Based on the relatively intact structure of the intestinal mucosal glands and marked alleviation of inflammatory cell infiltration, EGT showed a protective effect (Wang, Xie, et al. 2024).

Since then, an increasing amount of evidence has been accumulating on the role that gut microbiota plays as one of the major regulators in the physiology of the host coordinating its immunity, maintaining homeostasis, as well as contributing to the progression of inflammatory bowel disease (Sartor 2006). Dysbiosis has been associated with ulcerative colitis (UC). In the current study, the alpha diversity of gut microbiota was significantly lower after the administration of DSS in mice, along with a decrease in both the Chao1 index and the Shannon index. However, the treatment of EGT reversed this drop in richness and evenness for microbes. Additionally, PCoA showed that EGT obviously changed the whole microbial community structure toward the healthy. The result suggests that EGT could mitigate DSS‐induced dysbiosis via increasing microbial diversity and ecological stability.

The overall bacterial community structure was determined at the phylum level; our results showed that the intestinal microbiota had undergone substantial alteration after DSS exposure with an increased proportion of *Proteobacteria* while decreased proportions of *Firmicutes* were observed. The latter phylum is widely recognized as a key component of the gut microbiota, with established protective roles against IBD (Tsai et al. 2025). In contrast, *Proteobacteria* are Gram‐negative, a classification defined by the presence of an outer membrane. This membrane contains LPS, a potent trigger of gut dysbiosis and intestinal inflammation (Saedi et al. 2025). After EGT treatment, *Firmicutes* showed significantly increased relative abundance while *Proteobacteria* and *Verrucomicrobia* were significantly decreased. On a genus taxonomic level, the results showed that DSS treatment resulted in a significant increase in the relative abundance of *Escherichia‐Shigella*, a microbiome signature strongly linked to the gut dysbiosis (Li et al. 2022). EGT intervention effectively restored the balance of the gut microbiota by suppressing the proliferation of *Escherichia‐Shigella* and concurrently enriching beneficial taxa such as *Lachnospiraceae_NK4A136*_group. Members of *Lachnospiraceae* are generally considered important short‐chain fatty acid‐producing bacteria, especially butyrate‐producing taxa, which may contribute to epithelial energy metabolism, mucosal barrier integrity, and immune homeostasis.

Thus, the enrichment of the *Lachnospiraceae NK4A136* group may contribute to the enhanced intestinal barrier function and decreased inflammatory responses seen in the present study (Zhang et al. 2017). These changes of microbiota components were also confirmed by LEfSe that showed *Escherichia‐Shigella* was a predominant genus in the DSS treated group, whereas the addition of EGT promoted the enrichment of some probiotic genera such as *Eubacterium*, *Oscillibacter*, or *Paramuribaculum*. Together, these findings suggest that the administration of EGT helps to maintain a healthy microbiota in DSS‐colitis through increasing some commensals and suppressing pathogens.

This study has several limitations. First, the in vitro experiments were performed only in Caco‐2 cells, which cannot fully reproduce the complex cellular composition and immune microenvironment of the intestinal mucosa. Second, although 100 μg/mL LPS induced a clear inflammatory response, this concentration also reduced cell viability; therefore, possible cytotoxic effects should be considered when interpreting the in vitro results. Third, the in vivo findings were obtained using a DSS‐induced acute colitis mouse model, which does not completely represent the chronic and relapsing characteristics of human IBD. Fourth, gut microbiota changes were analyzed using 16S rRNA sequencing, which provides taxonomic information but limited functional resolution. Therefore, the causal relationship between EGT‐induced microbial changes and colitis improvement remains to be further verified.

Future studies should include additional intestinal epithelial and immune cell models, lower‐dose inflammatory stimulation systems, chronic or relapsing colitis models, and microbiota‐depletion or fecal microbiota transplantation experiments to clarify whether the protective effects of EGT are microbiota‐dependent. Moreover, metagenomic, transcriptomic, and metabolomic approaches should be integrated to determine whether EGT regulates microbial metabolic pathways, such as short‐chain fatty acid production, bile acid metabolism, or oxidative stress‐related pathways. These studies will help further elucidate the translational potential of EGT as a dietary functional ingredient for intestinal inflammatory diseases.

## 5 Conclusions

In conclusion, this study demonstrated that EGT alleviated experimental colitis through multiple coordinated effects, including attenuation of inflammatory responses, improvement of intestinal epithelial barrier‐related markers, reduction of colonic histopathological injury, and modulation of gut microbiota composition. In vitro, EGT reduced LPS‐induced inflammatory cytokine production and partially restored the expression and distribution of tight junction proteins in Caco‐2 cells. In vivo, EGT supplementation mitigated DSS‐induced clinical symptoms, inflammatory responses, immune activation, and colon tissue damage. Moreover, EGT partially reshaped the DSS‐disrupted gut microbiota, with high‐dose EGT promoting the enrichment of *Lachnospiraceae NK4A136* group and *Enterorhabdus*. These findings suggest that EGT may serve as a promising functional food ingredient for the prevention or adjunctive management of intestinal inflammation. Nevertheless, further studies using chronic colitis models, functional microbiome analysis, and metabolomic validation are needed to clarify the precise mechanisms and translational value of EGT.

## Author Contributions

**Yucong Wang:** data curation. **Lin Wang:** conceptualization, writing – review and editing. **Yao Song:** data curation. **Zhixin Xie:** writing – original draft. **Xihan Xu:** data curation. **Rongxu Liu:** conceptualization. **Jianchun Han:** conceptualization.

## Funding

This research was funded by Scientific Institution Basal Research Fund of Heilongjiang Province (Youth Project) (CZKYF2024‐1‐C012) and National Undergraduate Innovation and Entrepreneurship Training Program (202510224018S) and “The APC was funded by Heilongjiang Provincial Department of Finance”.

## Conflicts of Interest

The authors declare no conflicts of interest.

## Supporting information


**Table S1:** RT‐qPCR primer sequences.

## Data Availability

The data that support the findings of this study are available from the corresponding author upon reasonable request.
