# Supplementary material for: A Multi‐Target Approach to Colitis: Ergothioneine Promotes Recovery by Restoring the Intestinal Barrier, Reducing Inflammation, and Remodeling the Microbiota
Source: Food Sci Nutr. 2026 Sep 10;14(9):e72294. doi: 10.1002/fsn3.72294 (PMC13562916; doi:10.1002/fsn3.72294)
Supplement: Supplementary file 1 [file FSN3-14-e72294-s001.docx]

Table S1 RT-qPCR primer sequences

| Gene | Forward Primer | Reverse Primer |
| --- | --- | --- |
| ZO-1 | GGATGTTTATCGCATTGTA | AAGAGCCCAGTTTTCCATTGTA |
| Occludin | TCTAGGACGCAGCAGATTGG | TGGACTTTCAAGAGGCCTGG |
| Claudin-1 | AGTTAGGAGCCTTGATGCCG | GCACAGGGAGTAGGATACGC |
| TNF-α | CCCTCACACTCAGATCATCTTCT | CTACGACGTGGGCTACAG |
| IL-1β | TGACGGACCCCAAAAGATGA | TCTCCACAGCCACAATGAGT |
| IL-6 | TGAAGCACCCACCAATACAA | CCAACCTCAGAAAGCAGCTT |
| GAPDH | TGGAGAAACCTGCCAAGTATGA | TGGAAGAATGGGAGTTGCTGT |
